# Longitudinal evaluation of HIV-1 LAg incidence assay and proficiency panel stability for external quality assurance

**DOI:** 10.1128/spectrum.03584-25

**Published:** 2026-04-10

**Authors:** Clara Di Germanio, Wes Rountree, Eduard Grebe, Mars Stone, Aditi Sanghavi, Brendan G. Balasko, Jahnavi Bhaskar, Patricia G. Villaflor, Paul M. Hargarten, Marek Poniewierski, Sylvester Hood, Cassandra G. Porth, Thomas N. Denny, Michael P. Busch

**Affiliations:** 1 Vitalant Research Institute 166672, San Francisco, California, USA; 2 Department of Laboratory Medicine, University of California 8785, San Francisco, California, USA; 3 Duke Human Vaccine Institute, Duke University 3065, Durham, North Carolina, USA; Naturwissenschaftliches und Medizinisches Institut an der Universitat Tubingen, Reutlingen, Germany

**Keywords:** limited antigen avidity assay, quality assurance, recency, HIV

## Abstract

Accurate HIV incidence estimates are essential for monitoring epidemic trends and evaluating prevention efforts. The limiting antigen (LAg) avidity enzyme immunoassay is the most widely used method to classify recent versus long-term HIV infections. The External Quality Assurance Oversight Laboratory (EQAPOL), in collaboration with Vitalant Research Institute, conducts a global External Quality Assurance (EQA) program to evaluate laboratory performance using commercial LAg kits. We assessed (i) the stability of calibrators and reagents from Sedia and Maxim kits over their shelf life, and (ii) the longitudinal consistency of EQA panel samples tested across nine proficiency rounds (2018–2023). Mixed-effects models were used to detect trends in optical density (OD) and normalized OD (ODn) values over time. No significant trends in OD or ODn were observed for either kit, and EQA panel samples showed stable reactivity across all rounds. Minor ODn shifts did not affect recency classification. Sedia kits consistently yielded slightly higher ODn values than Maxim kits, consistent with prior reports. These findings confirm the long-term reliability of both LAg assay kits and EQA panel materials, supporting the robustness of EQAPOL’s quality assurance framework for accurate global HIV incidence surveillance.

**IMPORTANCE:**

Reliable estimation of HIV incidence depends on accurate classification of recent versus long-term infections, which in turn requires consistent assay performance and robust quality assurance. The External Quality Assurance Oversight Laboratory LAg External Quality Assurance program provides longitudinal monitoring of laboratory proficiency for two widely used commercial assays. By demonstrating the long-term consistency of calibrators, reagents, and quality control panels across multiple production lots and testing rounds, this study confirms the robustness of the global limiting antigen testing framework. These results strengthen confidence in the comparability of HIV incidence data across surveillance networks and geographic regions, supporting evidence-based decision-making for HIV prevention programs.

## INTRODUCTION

Epidemiological tracking of HIV and assessing the impact of public health interventions, such as education and pre-exposure prophylaxis, rely on accurate estimates of incidence (1). Cross-sectional HIV incidence testing, which relies on identifying recent infections among HIV-positive individuals, can help avoid the expense and certain biases of longitudinal studies (2–5). The External Quality Assurance Oversight Laboratory (EQAPOL) at the Duke Human Vaccine Institute (6), in partnership with Vitalant Research Institute (VRI), evaluates the proficiency of laboratories supported by NIAID/DAIDS and the CDC in performing the most widely employed HIV-1 incidence assay, the limiting antigen (LAg) avidity enzyme immunoassay (7). This assay classifies infections as recently acquired or long-term for HIV surveillance and cross-sectional incidence estimation (8).

The EQAPOL LAg Incidence Assay External Quality Assurance (EQA) program assesses laboratory performance over time, including the qualitative and quantitative accuracy, precision, and consistency of LAg testing using panels of well-characterized human plasma samples. It also monitors assay performance across different manufacturers, kit lots, and time. Twice a year, the program provides participating laboratories with an external proficiency (EP) panel to test using commercially available LAg kits from Sedia Biosciences and Maxim Biomedical (7).

Both LAg assays generate an optical density (OD) reading, which is normalized to an ODn value by dividing the measured OD of the tested samples by the OD of a kit calibrator (CAL) sample. Each manufacturer supplies a calibrator in the kit to minimize variability due to laboratory procedures, lot-to-lot inconsistencies, and potential reagent degradation over time. Despite these safeguards, little is known about how well the reagents, calibrators, and EP samples maintain their integrity throughout their shelf life and across multiple proficiency testing rounds, raising concerns about the long-term reliability of assay results.

There were two objectives for this study. First, we investigated whether the calibrators and controls of the commercially available Maxim and Sedia LAg kits appropriately control for any changes in reactivity over the full lifespan of the kits. This analysis was considered the kit stability assessment. Second, we evaluated if each panel sample’s ODn values in the EP program were stable over nine rounds of EQA testing (EP4–EP12). This analysis was considered the EQA longitudinal sample testing assessment. Given the importance of the LAg assay in HIV incidence surveillance, understanding the long-term reliability of reagents is crucial.

## MATERIALS AND METHODS

### Assay and testing schedule

For the kit stability analysis, the External Quality Assurance Oversight Laboratory at Duke and Core Immunology Laboratory at VRI performed the LAg assays in parallel according to the manufacturer’s instructions. Single lots of LAg kits, purchased at the same time, were used for both the Sedia (Lot #D0606; expiration: 22 June 2021) and Maxim (Lot #J8597; expiration: 15 September 2021) assays at both testing sites. Samples were tested in replicates of six every 3 months over the course of the 18-month in-date periods on both the Sedia and Maxim kits at both laboratories. Two additional time points accounted for 1 month post-expiration and 3 months post-expiration of the kits and were tested at VRI (Table S1).

For the longitudinal EQA samples analysis, EP results collected over time (nine rounds between August 2018 and November 2023) from the participating sites were used (Table S2). Briefly, blinded external proficiency panels were sent to up to 18 testing sites across North America, Africa, Asia, South America, and Europe. Each panel included 10 plasma samples: three blinded HIV-1-positive samples (included in replicate) representing recent and long-term infections with ODn values across the assay’s dynamic range and one HIV-negative control. Participating laboratories followed the manufacturer’s protocols for testing and completed a survey detailing kit manufacturer, lot number, testing procedures, and technician experience (7).

For the LAg assay, specimens were diluted 1:100 in diluent, and 100 μL was added to appropriate wells of antigen-coated plates and incubated for 60 min at 37°C. Plates were washed four times with 1× wash buffer to remove unbound antibodies. A pH 3.0 buffer was added to each well and incubated for 15 min at 37°C to dissociate low-avidity antibodies. Washed plates were incubated with anti-human IgG peroxidase (30 min at 37°C), then washed and incubated with tetramethyl benzidine substrate (15 min at 25°C). Color development was stopped by the addition of 100 μL/well of Stop Solution. The OD was read at 450 nm with 650 nm as a reference using a spectrophotometer. Raw OD for each specimen was normalized using the calibrator (CAL) OD on each plate as a ratio, such that ODn = OD of specimen/median OD of CAL. Negative and positive controls were tested on every plate and determined to be within range to confirm the validity of the plate. For both manufacturers, the ODn cutoff value of 1.5 is used to establish a recency classification.

### Samples

The EQAPOL LAg Incidence Assay EQA program provides participating sites with blinded panels of plasma samples derived from HIV-positive and HIV-negative blood donors identified through routine blood screening. Samples from HIV-infected donors were categorized as recent or long-term HIV infections based on replicate LAg testing at VRI.

For the kit stability assessment, human plasma samples from HIV-positive and HIV-negative donors used in previous rounds of EQA testing (EPs 3–7) (7) and that had therefore been well characterized through multiple rounds of testing with assays from both manufacturers, were selected as summarized in Table S3. The selected samples had ODn values that spanned the dynamic range of the assay and fell into the following categories: 0.1–0.5 ODn (recent low), 0.5–1.5 ODn (recent high), and 1.5 ODn or higher (long term). The panel also included samples near the 1.5 ODn threshold that determines recent and long-term classification. A sufficient number of aliquots for the entire study were prepared in advance and frozen.

For the EQA longitudinal sample testing assessment, results collected during routine EPs 4–12 for HIV-positive samples with at least four time points were included (Table S4). Unlike the kit stability assessment, this analysis evaluated the sample stability/consistency based on ODn values from the end of 2018 through the beginning of 2024.

### Statistical analysis

To determine if the Maxim and Sedia kit calibrators maintained consistent OD and ODn values for the life of the kits, tests for linear trends over the 26-month testing period were performed using Proc Mixed in SAS 9.4. There were models for OD and ODn values to estimate and compare slopes, separately for the Maxim and Sedia assays. The HIV-negative sample was not included in the analysis. The OD or ODn values were the outcome variable with fixed effects for an interaction of kit type by sample ID and an interaction of kit type by sample ID by days, with a random effect for laboratory. We analyzed the OD and ODn values obtained over time on the same samples through and beyond kit expiration dates to see if there were linear trends in reactivity with kit aging.

An analysis of the stability of donor samples over time in the LAg EP Program was also performed using Proc Mixed in SAS 9.4. There were models for ODn values (because these, but not ODs, are used for the grading, and the kit stability assessment evaluates the relationship of the calibration to ODn values) to estimate slopes and compare the slopes for the Maxim and Sedia assays. The ODn values were the outcome variable with fixed effects for an interaction of kit type by sample ID and an interaction of kit type by sample ID by days, with a random effect for laboratory by donor.

For these analyses, the alpha level was set at 0.05 for all significance testing, with no adjustment for multiple comparisons. This allows for the most sensitive testing of linear trends in these data.

## RESULTS

### Kit stability assessment

**Fig 1:**
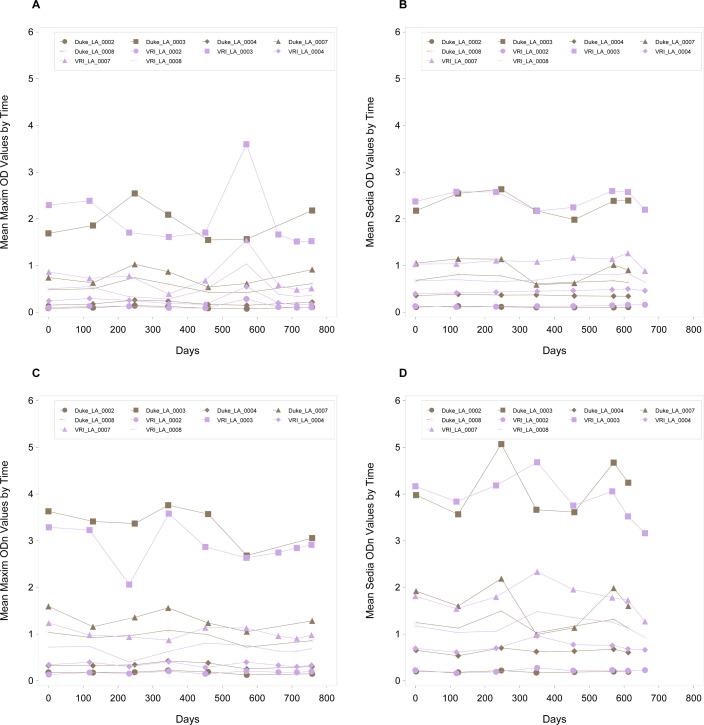
Plot of OD and ODn values: kit and lab comparisons from Sedia and Maxim LAg kits, across the two different testing sites (VRI and Duke). (**A**) Plot of OD values by time for maxim kits. (**B**) Plot of OD values over time for sedia kits. (**C**) Plot of ODn values by time for maxim kits. (**D**) Plot of ODn values by time for sedia kits.

There was no statistical evidence of any linear trends of OD values for the samples tested. There were no slopes significantly different from zero for either kit manufacturer, nor was there a significant difference between the kits. Reactivity was stable over the timespan of the study (Fig. 1A and B; Table 1).

**TABLE 1:** Linear trends in OD values over time by sample and kit manufacturer, including daily slope estimates and between-kit comparisons

Sample	Kit type	Daily OD slope	Daily OD slope *P* value	OD slope comparison (Maxim-Sedia)	OD slope comparison *P* value
LA_0002	Maxim	−0.0000056	0.9574		
	Sedia	0.0000197	0.7773	−0.0000253	0.8403
LA_0003	Maxim	−0.0001183	0.2574		
	Sedia	−0.0001092	0.1171	−0.0000091	0.9422
LA_0004	Maxim	−0.0000020	0.9848		
	Sedia	0.0000670	0.3360	−0.0000690	0.5821
LA_0007	Maxim	−0.0000462	0.6578		
	Sedia	−0.0001060	0.1283	0.0000598	0.6335
LA_0008	Maxim	−0.0000350	0.7370		
	Sedia	0.0000158	0.8198	−0.0000509	0.6847

However, there was statistical evidence of a slight linear decrease in ODn values for samples LA_0003 (both kits) and LA_007 (Maxim only). LA_0003 was a long-term sample, and the average ODn per time point was always above 2.0 (within the range for the long-term category), but it underwent the most significant decline using the Maxim kit, with a daily reduction of −0.00075 ODn. This translates into a decline of 0.57 ODn units over the 758 days, with a starting value of approximately 3.5 ODn and a final value of approximately 3.0 ODn. LA_0007 was a near-cutoff sample that had an average ODn ranging from 0.9 to 1.6 for both kits over time. The recent samples (LA_0002 and LA_0004) were always within the range of their category. The near-cutoff samples occasionally switched classifications, i.e., crossed the 1.5 ODn threshold and alternated between recent and long term. There were no significant differences in slopes for the two kits, which indicates that the ODn values were rather stable over the timespan of the kit stability study which went beyond the manufacture stipulated expiration dates (Fig. 1C and D; Table 2).

**TABLE 2:** Linear trends in ODn values over time by sample and kit manufacturer, including daily slope estimates and between-kit comparisons

Sample	Kit type	Daily ODn slope	Daily ODn slope *P* value	ODn slope comparison (Maxim-Sedia)	ODn slope comparison *P* value
LA_0002	Maxim	0.0000249	0.8007		
	Sedia	0.0000509	0.7367	−0.0000260	0.8854
LA_0003	Maxim	−0.0007522	<0.0001		
	Sedia	−0.0004972	0.0011	−0.0002551	0.1582
LA_0004	Maxim	−0.0000151	0.8786		
	Sedia	0.0000810	0.5931	−0.0000960	0.5950
LA_0007	Maxim	−0.0003204	0.0012		
	Sedia	−0.0002768	0.0682	−0.0000436	0.8094
LA_0008	Maxim	−0.0001796	0.0691		
	Sedia	−0.0000228	0.8803	−0.0001568	0.3856

### Calibrator controls

**Fig 2:**
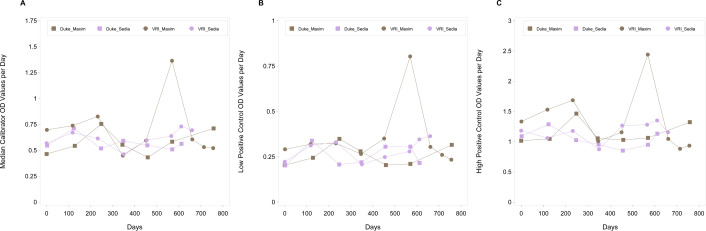
Plot of controls (calibrator, low, and high controls) over time. (**A**) Plot of meidan calibrator OD values by day: LAg calibrator study. (**B**) Plot of low positive control OD values by day: LAg calibrator study. (**C**) Plot of high positive control OD values by day: LAg calibrator study.

During the June 2021 testing time point with the Sedia kit, the median OD values for the calibrator, low-positive control, and high-positive control fell outside the manufacturer-specified acceptability ranges, which would render the assay run invalid according to the manufacturer’s protocol (Fig. 2). This occurrence was observed at only one laboratory. Although the run would normally be excluded from analysis, we evaluated the data to assess calibrator performance and found that OD normalization remained effective, with all sample ODn values falling within their expected ranges.

### Performance across kit manufacturers

**Fig 3:**
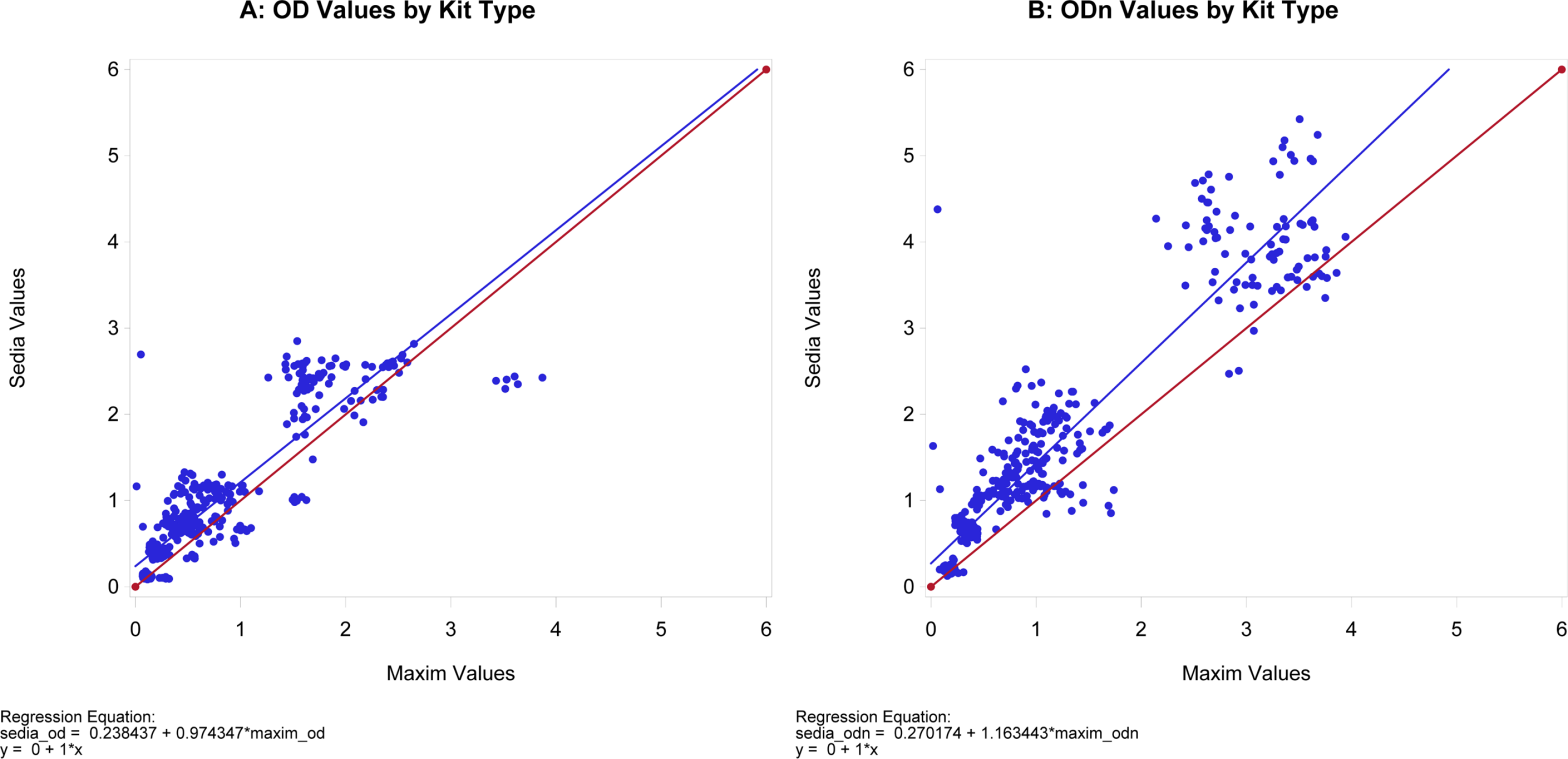
Comparison of Maxim and Sedia OD and ODn values. Panel **A** shows OD readings, and panel **B** shows ODn readings. The blue line is the linear regression line, and the red line shows the slope if the two kits had produced equivalent results.

Figure 3 shows the raw OD and normalized ODn values for the two kit types on the same samples. Consistent with findings from previous studies, these graphs indicate that the OD values are more similar than the ODn values between the two kits, indicating that differences are exacerbated by differences in calibrators. ODn values from Sedia kits tended to be higher than ODn values produced using the Maxim kits, as previously reported (7, 9, 10).

### EQA longitudinal samples stability assessment

**Fig 4:**
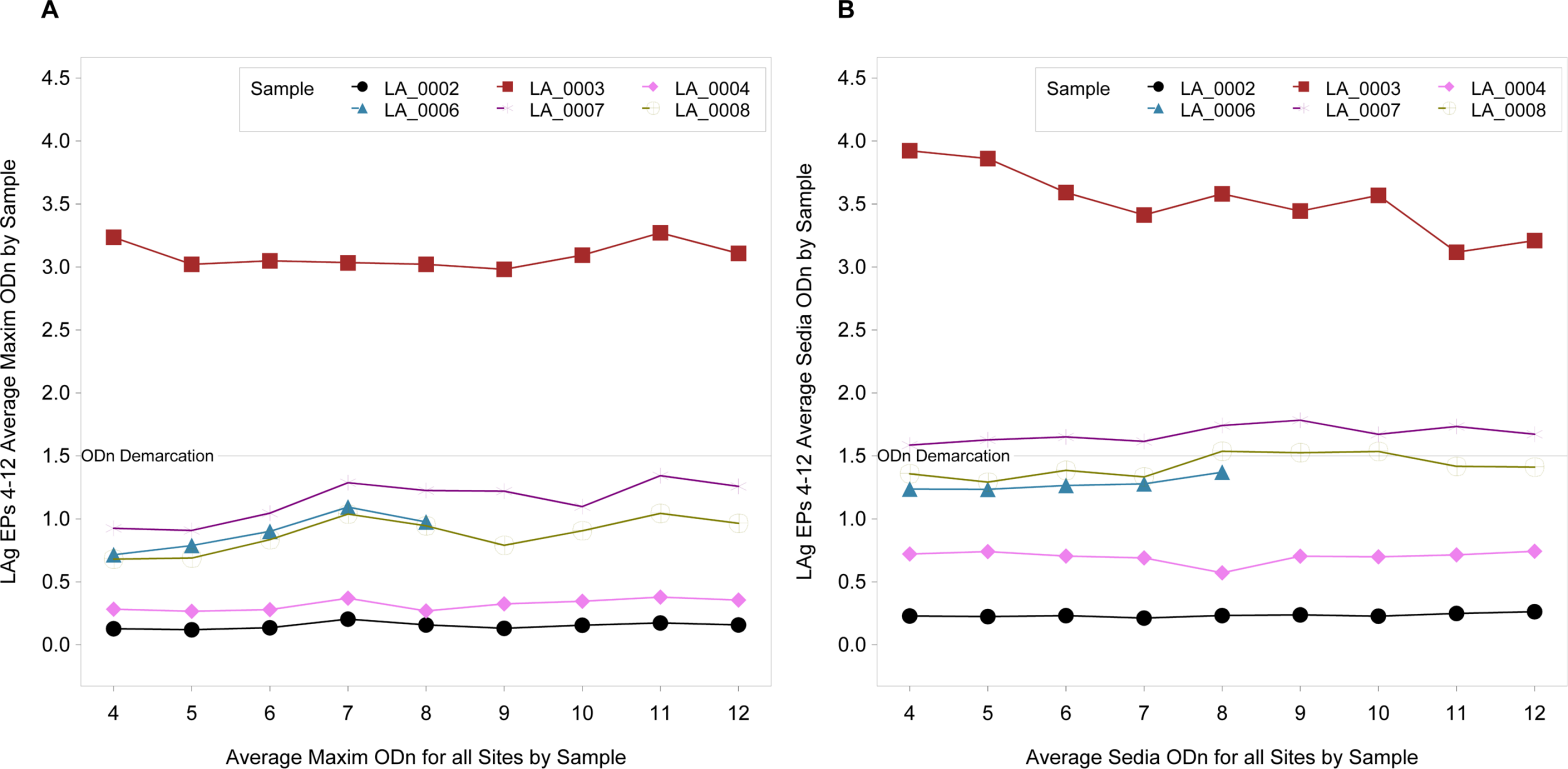
Average longitudinal ODn of samples for EPs 4–12. (**A**) Average maxim ODn by sample for EPs 4-12. (**B**) Average sedia ODn by sample for EPs 4-12.

To address the question of the panel’s sample stability over time, the results of six samples tested between the fall of 2018 and the fall of 2023 were analyzed. Five of the six samples were used in all nine EPs, and one sample (LA_0006) contributed to five EPs (4–8). Figure 4A and B show the sample averages at each EP for either the Sedia or Maxim kit, while Table S5 includes the relative 95% confidence intervals. Table 3 shows the slope estimates for each kit by sample over the nine EPs. There are eight slopes significantly different from zero, with only one of these slopes being negative indicating a decrease over time for LA_0003. This was a long-term sample with an ODn value well above 3, and the model estimated decline over nine EPs was only −0.76 in ODn value, which would still be well above the 1.5 threshold. Table 4 provides a comparison of the slopes between kit types by sample. Only LA_0002 is not significantly different between the kit types, and the independent kit slopes are not significant either, which is not unexpected given this was the recent (low) sample. The kit slopes differ, but the slope values are very low, and samples do not change categories over time. Table 5 shows that the mean ODn values for Sedia kits are all significantly higher for the six samples than the Maxim ODn values.

**TABLE 3:** Linear trends of ODn values across nine EP rounds for Maxim and Sedia LAg kits

Kit	Sample	Slope estimate	Slope 95% CI	ODn change since EP4	*P* value
Maxim	LA_0002	0.0035	0.0035 (−0.0028, 0.0097)	0.028	0.2727
Sedia	LA_0002	0.0043	0.0043 (-0.0016, 0.0102)	0.035	0.1513
Maxim	LA_0003	−0.0023	−0.0023 (−0.0116, 0.0070)	−0.018	0.6285
Sedia	LA_0003	−0.0946	−0.0946 (−0.1036, −0.0856)	−0.757	<0.0001
Maxim	LA_0004	0.0115	0.0115 (0.0057, 0.0173)	0.092	<0.0001
Sedia	LA_0004	−0.0003	−0.0003 (−0.0059, 0.0053)	−0.002	0.9137
Maxim	LA_0006	0.0741	0.0741 (0.0513, 0.0969)	0.593	<0.0001
Sedia	LA_0006	0.0277	0.0277 (0.0067, 0.0487)	0.222	0.0098
Maxim	LA_0007	0.0468	0.0468 (0.0368, 0.0568)	0.374	<0.0001
Sedia	LA_0007	0.0166	0.0166 (0.0071, 0.0261)	0.133	0.0006
Maxim	LA_0008	0.0346	0.0346 (0.0246, 0.0446)	0.277	<0.0001
Sedia	LA_0008	0.0183	0.0183 (0.0088, 0.0278)	0.146	0.0002

**TABLE 4:** Between-kit comparison of mean longitudinal ODn slopes across nine EP rounds

Sample	Maxim slope	Sedia slope	Maxim-Sedia (95% CI)	*P* value
LA_0002	0.0035	0.0043	−0.0008 (−0.0095, 0.0078)	0.8507
LA_0003	−0.0023	−0.0946	0.0923 (0.0793, 0.1054)	<0.0001
LA_0004	0.0115	−0.0003	0.0118 (0.0037, 0.0199)	0.0044
LA_0006	0.0741	0.0277	0.0464 (0.0150, 0.0778)	0.0037
LA_0007	0.0468	0.0166	0.0302 (0.0163, 0.0441)	<0.0001
LA_0008	0.0346	0.0183	0.0163 (0.0024, 0.0303)	0.0217

**TABLE 5:** Comparison of mean ODn values between Maxim and Sedia LAg kits across nine EP rounds

Sample	Maxim mean	Maxim SE	Sedia mean	Sedia SE	Maxim-Sedia (95% CI)	*P* value
LA_0002	0.141	0.030	0.232	0.029	−0.0903 (−0.1169, −0.0638)	<0.0001
LA_0003	3.099	0.032	3.591	0.031	−0.4918 (−0.5332, −0.4505)	<0.0001
LA_0004	0.328	0.029	0.699	0.029	−0.3711 (−0.3965, −0.3457)	<0.0001
LA_0006	1.042	0.039	1.322	0.039	−0.2795 (−0.3540, −0.2050)	<0.0001
LA_0007	1.117	0.032	1.685	0.031	−0.5682 (−0.6106, −0.5257)	<0.0001
LA_0008	0.833	0.032	1.410	0.031	−0.5774 (−0.6198, −0.5349)	<0.0001

## DISCUSSION

A core objective of the EQAPOL program is to support global HIV surveillance by developing and implementing robust proficiency testing to identify and mitigate sources of variability in HIV incidence assay results. Accurate and reliable HIV incidence estimates are essential for guiding public health strategies, optimizing resource allocation, and evaluating the effectiveness of prevention and treatment programs. In regions with high HIV prevalence, high-quality surveillance data are critical for deploying targeted interventions to reduce transmission rates (11).

This study addressed two key factors that could influence the reliability of HIV incidence testing over time: the stability of kit components, including calibrators, across their shelf life, and the consistency of EP samples across multiple testing rounds. These objectives reflect EQAPOL’s overarching mission to ensure precision and reproducibility across laboratories using different assay kits from multiple manufacturers.

Over an 18-month period, differences in OD and ODn were observed between the Sedia and Maxim kits. These differences were consistent with prior findings, including both single-laboratory evaluations and previous EQAPOL reports (9, 10). Importantly, no significant linear trends in ODn values were observed for individual samples when accounting for kit type, suggesting that degradation of the kit reagents including calibrators over time did not significantly impact assay results. This indicates that calibration procedures provided by the manufacturers are generally effective in preserving assay performance throughout the shelf life of the kits.

Having established kit and calibrator stability, the second objective focused on the long-term consistency of the EP panel’s samples distributed through the EQAPOL EQA program. Analysis of EP panels from rounds 4 through 12 revealed highly consistent ODn values across all samples, demonstrating minimal drift over time. While a few statistically significant trends were identified, these shifts were small and did not affect sample classification—except in one borderline case (sample LA_0008 with the Sedia kit), where ODn values hovered near the recency threshold. This sample was intentionally selected near the cutoff to evaluate assay sensitivity and is not indicative of a broader issue with sample stability.

Together, these findings support the long-term reliability of both the kit constituents, including calibrators, and the EP samples used in HIV incidence testing EQA programs. However, they also emphasize the importance of maintaining consistency in testing protocols. Switching between assay kits mid-study or across testing sites could introduce variability in OD or ODn values, potentially affecting incidence estimates or masking true epidemiological trends. Operator technique and laboratory-specific practices may further contribute to such variability and should be considered during data interpretation.

The EQAPOL LAg External Quality Assurance program remains a vital resource for the global network of laboratories performing HIV incidence testing. By providing regular quality assessments, offering technical remediation, and facilitating standardized training, the program enhances laboratory proficiency and ensures high-quality data generation, per the WHO guidelines on HIV testing (12). This is especially important in field settings where resource constraints and environmental variability can compromise assay performance. Continued investment in standardized protocols, personnel training, and external quality assurance monitoring is essential to sustain accurate and actionable HIV incidence estimates. EQAPOL’s efforts contribute meaningfully to the global HIV response by reinforcing the reliability of surveillance data, ultimately supporting universal testing, treatment, and suppression strategies aimed at reducing new infections and advancing public health. Finally, the EQAPOL program supports implementation science by enabling reliable measurement of recent HIV transmission in real-world settings, thereby informing the population-level impact of prevention and treatment intervention effectiveness, and program scale-up aimed at sustained transmission reduction and HIV cure strategies.

## SUPPLEMENTAL MATERIAL

Supplemental tables spectrum.03584-25-s0001.docx Tables S1 to S5.

ASM does not own the copyrights to Supplemental Material that may be linked to, or accessed through, an article. The authors have granted ASM a non-exclusive, world-wide license to publish the Supplemental Material files. Please contact the corresponding author directly for reuse.
