# Supplementary material for: Longitudinal evaluation of HIV-1 LAg incidence assay and proficiency panel stability for external quality assurance
Source: Microbiol Spectr. 2026 Apr 10;14(5):e03584-25. doi: 10.1128/spectrum.03584-25 (PMC13141909; doi:10.1128/spectrum.03584-25)
Supplement: Supplemental tables — Tables S1 to S5. [file spectrum.03584-25-s0001.docx]

Supplemental Table 1. LAg Kits Stability Study Testing Timepoints

| **Testing Date** | **Kits Tested^#^** | **Time Post Expiration** |
| --- | --- | --- |
| November 18, 2019 | Sedia + Maxim | - |
| March 23, 2020 | Sedia + Maxim | - |
| July 23, 2020 | Sedia + Maxim | - |
| October 26, 2020 | Sedia + Maxim | - |
| February 15, 2021 | Sedia + Maxim | - |
| June 7, 2021 | Sedia + Maxim | - |
| July 26, 2021 | Sedia | 1 month post Sedia |
| September 7, 2021 | Sedia* + Maxim | 3 months post Sedia |
| October 18, 2021 | Maxim* | 1 month post Maxim |
| December 13, 2021 | Maxim* | 3 months post Maxim |

^#^ Sedia expiration: June 22, 2021; Maxim expiration: September 15, 2021

*tested at VRI only

Supplemental Table 2. EPs Panels Testing Timepoints

| **Testing Date** | **Kits Tested** | **# of participating sites** |
| --- | --- | --- |
| August 2018 | Sedia + Maxim | 18 |
| January 2019 | Sedia + Maxim | 18 |
| August 2019 | Sedia + Maxim | 18 |
| January 2020 | Sedia + Maxim | 17 |
| June 2021 | Sedia + Maxim | 15 |
| January 2022 | Sedia + Maxim | 14 |
| October 2022 | Sedia + Maxim | 14 |
| April 2023 | Sedia + Maxim | 12 |
| November 2023 | Sedia + Maxim | 12 |

Supplemental Table 3. Human Serum Samples for kit stability evaluation

|  | Reagents stability sample ID | **Sample Type** | **Expected Categorization** |
| --- | --- | --- | --- |
|  | Negative Human Plasma | Negative | < 0.2 |
|  | LA_0002 | Recent (Low) | 0.2 – 0.5 |
|  | LA_0003 | Long Term | > 2.0 |
|  | LA_0004 | Recent (High) | 0.5 – 1.0 |
|  | LA_0007 | Near Cutoff | 1.0 – 2.0 |
|  | LA_0008 | Near Cutoff | 1.0 – 2.0 |

Supplemental Table 4. Human Serum Samples for EP panel longitudinal stability evaluation

|  | EP 4-12 sample ID | **Sample Type** | **Expected Categorization** |
| --- | --- | --- | --- |
|  | LA_0002 | Recent (Low) | 0.2 – 0.5 |
|  | LA_0003 | Long Term | > 2.0 |
|  | LA_0004 | Recent (High) | 0.5 – 1.0 |
|  | LA_0006 | Near Cutoff | 1.0 – 2.0 |
|  | LA_0007 | Near Cutoff | 1.0 – 2.0 |
|  | LA_0008 | Near Cutoff | 1.0 – 2.0 |

Supplemental Table 5. 95% Confidence intervals for sample averages at each EP for either the Sedia or Maxim kit

| **EP** | **Sample** | **Kit Type** | **95% Confidence Interval** |
| --- | --- | --- | --- |
| 4 | LA_0002 | Maxim | 0.13 (0.20, 0.04) |
| 5 | LA_0002 | Maxim | 0.12 (0.17, 0.06) |
| 6 | LA_0002 | Maxim | 0.14 (0.22, 0.04) |
| 7 | LA_0002 | Maxim | 0.20 (0.31, 0.08) |
| 8 | LA_0002 | Maxim | 0.16 (0.26, 0.04) |
| 9 | LA_0002 | Maxim | 0.13 (0.19, 0.06) |
| 10 | LA_0002 | Maxim | 0.16 (0.26, 0.05) |
| 11 | LA_0002 | Maxim | 0.17 (0.22, 0.12) |
| 12 | LA_0002 | Maxim | 0.16 (0.19, 0.11) |
| 4 | LA_0003 | Maxim | 3.24 (3.94, 2.52) |
| 5 | LA_0003 | Maxim | 3.02 (3.81, 2.22) |
| 6 | LA_0003 | Maxim | 3.05 (3.89, 2.20) |
| 7 | LA_0003 | Maxim | 3.03 (3.91, 2.15) |
| 8 | LA_0003 | Maxim | 3.02 (4.16, 1.87) |
| 9 | LA_0003 | Maxim | 2.98 (3.85, 2.10) |
| 10 | LA_0003 | Maxim | 3.09 (4.33, 1.85) |
| 11 | LA_0003 | Maxim | 3.27 (4.12, 2.42) |
| 12 | LA_0003 | Maxim | 3.11 (4.15, 2.06) |
| 4 | LA_0004 | Maxim | 0.28 (0.36, 0.19) |
| 5 | LA_0004 | Maxim | 0.27 (0.36, 0.17) |
| 6 | LA_0004 | Maxim | 0.28 (0.41, 0.14) |
| 7 | LA_0004 | Maxim | 0.37 (0.53, 0.20) |
| 8 | LA_0004 | Maxim | 0.27 (0.39, 0.13) |
| 9 | LA_0004 | Maxim | 0.32 (0.45, 0.19) |
| 10 | LA_0004 | Maxim | 0.35 (0.54, 0.14) |
| 11 | LA_0004 | Maxim | 0.38 (0.49, 0.26) |
| 12 | LA_0004 | Maxim | 0.36 (0.42, 0.28) |
| 4 | LA_0006 | Maxim | 0.72 (1.02, 0.40) |
| 5 | LA_0006 | Maxim | 0.79 (1.04, 0.52) |
| 6 | LA_0006 | Maxim | 0.90 (1.28, 0.51) |
| 7 | LA_0006 | Maxim | 1.09 (1.47, 0.71) |
| 8 | LA_0006 | Maxim | 0.98 (1.29, 0.65) |
| 4 | LA_0007 | Maxim | 0.92 (1.32, 0.52) |
| 5 | LA_0007 | Maxim | 0.91 (1.28, 0.53) |
| 6 | LA_0007 | Maxim | 1.05 (1.42, 0.66) |
| 7 | LA_0007 | Maxim | 1.29 (1.52, 1.05) |
| 8 | LA_0007 | Maxim | 1.23 (1.59, 0.85) |
| 9 | LA_0007 | Maxim | 1.22 (1.90, 0.53) |
| 10 | LA_0007 | Maxim | 1.10 (1.63, 0.55) |
| 11 | LA_0007 | Maxim | 1.34 (1.70, 0.98) |
| 12 | LA_0007 | Maxim | 1.26 (1.72, 0.78) |
| 4 | LA_0008 | Maxim | 0.68 (1.10, 0.25) |
| 5 | LA_0008 | Maxim | 0.69 (0.92, 0.45) |
| 6 | LA_0008 | Maxim | 0.83 (1.24, 0.42) |
| 7 | LA_0008 | Maxim | 1.04 (1.26, 0.80) |
| 8 | LA_0008 | Maxim | 0.94 (1.18, 0.70) |
| 9 | LA_0008 | Maxim | 0.79 (1.11, 0.46) |
| 10 | LA_0008 | Maxim | 0.91 (1.38, 0.43) |
| 11 | LA_0008 | Maxim | 1.04 (1.40, 0.68) |
| 12 | LA_0008 | Maxim | 0.96 (1.36, 0.55) |
| 4 | LA_0002 | Sedia | 0.23 (0.28, 0.16) |
| 5 | LA_0002 | Sedia | 0.22 (0.28, 0.15) |
| 6 | LA_0002 | Sedia | 0.23 (0.31, 0.15) |
| 7 | LA_0002 | Sedia | 0.21 (0.30, 0.11) |
| 8 | LA_0002 | Sedia | 0.23 (0.27, 0.18) |
| 9 | LA_0002 | Sedia | 0.24 (0.30, 0.17) |
| 10 | LA_0002 | Sedia | 0.23 (0.29, 0.15) |
| 11 | LA_0002 | Sedia | 0.25 (0.29, 0.20) |
| 12 | LA_0002 | Sedia | 0.26 (0.38, 0.14) |
| 4 | LA_0003 | Sedia | 3.92 (5.12, 2.72) |
| 5 | LA_0003 | Sedia | 3.86 (4.65, 3.06) |
| 6 | LA_0003 | Sedia | 3.59 (4.42, 2.75) |
| 7 | LA_0003 | Sedia | 3.41 (4.73, 2.09) |
| 8 | LA_0003 | Sedia | 3.58 (4.16, 2.99) |
| 9 | LA_0003 | Sedia | 3.44 (4.02, 2.86) |
| 10 | LA_0003 | Sedia | 3.57 (4.29, 2.84) |
| 11 | LA_0003 | Sedia | 3.12 (3.67, 2.56) |
| 12 | LA_0003 | Sedia | 3.21 (3.83, 2.57) |
| 4 | LA_0004 | Sedia | 0.72 (0.87, 0.56) |
| 5 | LA_0004 | Sedia | 0.74 (0.91, 0.56) |
| 6 | LA_0004 | Sedia | 0.70 (0.89, 0.51) |
| 7 | LA_0004 | Sedia | 0.69 (1.05, 0.32) |
| 8 | LA_0004 | Sedia | 0.57 (0.78, 0.36) |
| 9 | LA_0004 | Sedia | 0.70 (0.79, 0.61) |
| 10 | LA_0004 | Sedia | 0.70 (0.86, 0.53) |
| 11 | LA_0004 | Sedia | 0.71 (0.85, 0.57) |
| 12 | LA_0004 | Sedia | 0.74 (0.94, 0.54) |
| 4 | LA_0006 | Sedia | 1.24 (1.64, 0.82) |
| 5 | LA_0006 | Sedia | 1.23 (1.45, 1.01) |
| 6 | LA_0006 | Sedia | 1.27 (1.62, 0.90) |
| 7 | LA_0006 | Sedia | 1.28 (1.55, 0.99) |
| 8 | LA_0006 | Sedia | 1.37 (1.91, 0.82) |
| 4 | LA_0007 | Sedia | 1.59 (1.93, 1.23) |
| 5 | LA_0007 | Sedia | 1.63 (1.89, 1.35) |
| 6 | LA_0007 | Sedia | 1.65 (1.99, 1.30) |
| 7 | LA_0007 | Sedia | 1.62 (2.00, 1.22) |
| 8 | LA_0007 | Sedia | 1.74 (2.01, 1.46) |
| 9 | LA_0007 | Sedia | 1.78 (2.21, 1.35) |
| 10 | LA_0007 | Sedia | 1.67 (2.05, 1.28) |
| 11 | LA_0007 | Sedia | 1.73 (1.98, 1.48) |
| 12 | LA_0007 | Sedia | 1.67 (1.89, 1.44) |
| 4 | LA_0008 | Sedia | 1.36 (1.68, 1.02) |
| 5 | LA_0008 | Sedia | 1.29 (1.49, 1.08) |
| 6 | LA_0008 | Sedia | 1.39 (1.72, 1.04) |
| 7 | LA_0008 | Sedia | 1.33 (1.86, 0.79) |
| 8 | LA_0008 | Sedia | 1.54 (1.83, 1.24) |
| 9 | LA_0008 | Sedia | 1.53 (1.75, 1.30) |
| 10 | LA_0008 | Sedia | 1.54 (1.81, 1.25) |
| 11 | LA_0008 | Sedia | 1.42 (1.63, 1.20) |
| 12 | LA_0008 | Sedia | 1.41 (1.69, 1.13) |
